# GC-MS analysis and hepatoprotective activity of the *n*-hexane extract of *Acrocarpus fraxinifolius* leaves against paracetamol-induced hepatotoxicity in male albino rats

**DOI:** 10.1080/13880209.2016.1246575

**Published:** 2016-12-09

**Authors:** Eman A. Abd El-Ghffar, Heba A.S. El-Nashar, Omayma A. Eldahshan, Abdel Nasser B. Singab

**Affiliations:** a Department of Zoology, Faculty of Sciences, Ain Shams University, Abbassia, Cairo, Egypt;; b Department of Pharmacognosy, Faculty of Pharmacy, Ain Shams University, Abbassia, Cairo, Egypt

**Keywords:** Acetaminophen, ink cedar, gas chromatography mass spectrometry, liver dysfunction

## Abstract

**Context:** In Egypt, the burden of liver diseases is exceptionally high.

**Objective:** To investigate the components of the *n*-hexane extract of *Acrocarpus fraxinifolius* Arn. (Leguminosae) and its hepatoprotective activity against paracetamol (APAP)-induced hepatotoxicity in rats.

**Material and methods:** TRACE GC ultra gas chromatogaphic spectrometry was used for extract analysis. Thirty albino rats were divided into six groups (five rats in each). Group 1 was the healthy control; Groups 2 and 3 were healthy treated groups (250 and 500 mg/kg b.w. of the extract, respectively) for seven days. Group 4 was hepatotoxicity control (APAP intoxicated group). Groups 5 and 6 received APAP + extract 250 and APAP + extract 500, respectively.

**Results:** Chromatographic analysis revealed the presence of 36 components. Major compounds were *α-*tocopherol (18.23%), labda-8 (20)-13-dien-15-oic acid (13.15%), lupeol (11.93%), phytol (10.95%) and squalene (7.19%). In the acute oral toxicity study, the mortality rates and behavioural signs of toxicity were zero in all groups (doses from 0 to 5 g/kg b.w. of *A. fraxinifolius*). LD_50_ was found to be greater than 5 g/kg of the extract. Only the high dose (500 mg/kg b.w.) of extract significantly alleviated the liver relative weight (4.01 ± 0.06) and biomarkers, as serum aspartate aminotransferase (62.87 ± 1.41), alanine aminotransferase (46.74 ± 1.45), alkaline phosphatase (65.96 ± 0.74), lipid profiles (180.39 ± 3.51), bilirubin profiles (2.30 ± 0.06) and hepatic lipid peroxidation (114.20 ± 2.06), and increased body weight (11.58 ± 0.20), serum protein profile (11.09 ± 0.46) and hepatic total antioxidant capacity (23.78 ± 0.66) in APAP-induced hepatotoxicity in rats.

**Conclusion:** Our study proves the antihepatotoxic/antioxidant efficacies of *A. fraxinifolius* hexane extract.

## Introduction

Liver diseases are considered as a major public health problem around the world due to their potentiality to cause morbidity and mortality (Hasan & Khan 1997). About more than two million people in the world die annually from liver-related disorders (Bukhsh et al. 2014). Hepatitis viral infection, alcohols, drugs, industrial chemicals and pollutants are the major risk factors (Paraskevi & Ronald 2006).

Among drug-induced liver injury, paracetamol (acetaminophen, *N*-acetyl-*p*-aminophenol; APAP), is one of the most widely used hepatotoxic agents. It is safe at therapeutic doses, but causes liver failure in overdoses (Lewerenz et al. 2003). Paracetamol is used as an antipyretic drug (Ahmed & Khater 2001). It is safe at therapeutic doses, but at high doses, it produces acute liver failure and hepatic necrosis (Abraham 2005) and so, the American Association of Poison Control Center (AAPCC) announced that paracetamol toxicity leads to 401 deaths due to acute hepatic failure (Bronstein et al., 2009).

Oxidative stress plays a basic role in the pathogenesis of paracetamol-induced liver damage (Srinivasan et al. 2001). In overdose, paractamol is metabolized by liver cytochrome-450 enzymes into highly reactive metabolite *N*-acetyl-*p*-benzoquinoneimine (NAPQI) which causes depletion of 70% glutathione and subsequently oxidative stress (Bajt et al. 2001; Jaeschke et al. 2011; Gelotte et al. 2007). Also, NAPQI binds covalently to cellular proteins causing mitochondrial dysfunction, ATP depletion, lipid peroxidation, DNA damage and necrosis of parenchymal cells as well as hepatic necrosis (Jones et al. 2010). At the same time, damaged hepatocytes activate the liver’s innate immune response like Kupffer cells, natural killer cells, natural killer T cells. This results in producing pro-inflammatory mediators such as tumour necrosis factor-α, interferon-γ_2_ and interleukin-β causing liver injury (Kaplowitz et al. 2008).

Conventional drugs that are currently used in the treatment of liver diseases have poor efficacy with long-term use and potential toxic effects. Subsequently, scientists have been paying serious attention to alternative herbal therapies that have antioxidant properties with minimum side effects in the treatment of many diseases like liver diseases, to replace currently used drugs of doubtful efficacy and safety (Muthulingam et al. 2010). Medicinal plants play important roles in treating liver diseases. Lots of medicinal plants were used in the protection and treatment of various liver diseases (Saleem & Naseer 2014). Leaves of *Sapium sebiferum* (L.) Roxb (Euphorbiaceae) (Hussain et al. 2015) and *Malva sylvestris* L. (Malvaceae) (Hussain et al. 2014) showed strong hepatoprotective effects against paracetamol-induced liver injury. Leguminosae embraces several genera of reported hepatoprotective species (Huo et al. 2011; Rajendran et al. 2009; Arulkumaran et al. 2009; Rehman et al. 2015; Gamal El-Din et al. 2014; Azab et al. 2013).

*Acrocarpus fraxinifolius* Arn. (Leguminosae) is a member of the tribe Caesalpinieae (El-nashar et al. 2015). Mundani, shingle and pink cedar are its common names. It is a deciduous tree (heights of 30–60 m) and is considered as a part of tropical evergreen and sub-evergreen forests. This species is distributed naturally in countries such as India, Burma and China and is widely cultivated in Egypt. Traditionally, in Yanesha, boiled *A. fraxinifolius* bark is prepared as a drink and used for internal bruises (Valadeau et al. 2009). Recently, bioactive phenolics such as gallic acid, catechin, epicatechin and catechin gallate were identified from the bark extract by HPLC-DAD, and may be responsible for its radical scavenging activity (Rosales-castro et al. 2015). Total ethanol and aqueous ethanol extracts of the leaves exhibited high potencies as compared with metformin in decreasing the glucose level after four weeks. In addition, aforementioned extracts showed an *in vivo* antioxidant activity when compared to vitamin E (Abou Zeid et al. 2012).

This study was designed to investigate the hexane extract of *A. fraxinifolius* phytochemically for the first time as well as its hepatoprotective activity of the extract against APAP-induced hepatotoxicity in albino rats. At the same time, it investigated any deleterious effects caused by the consumption of *A. fraxinifolius* extract by healthy albino rats.

## Materials and methods

### Plant material

Leaves of *A. fraxinifolius* were collected from Giza Zoo Botanical Garden, in January 2013. They were kindly authenticated by Mrs. Tereize Labib, the taxonomy specialist in El-Orman Botanical Garden, Giza, Egypt. A voucher specimen (PHG-P-AF130) has been deposited at Pharmacognosy Department, Faculty of Pharmacy, Ain Shams University.

### Preparation of *n*-hexane extract

The intact air-dried plant material (2 kg) was comminuted to fine powder then soaked in 80% methanol for 4 days and filtered. The filtrate was completely evaporated *in vacuo* at ≈ 55 °C to achieve complete dryness. The dried residue was further successively fractionated with *n*-hexane. The combined *n*-hexane extracts were evaporated *in vacuo* until dryness to give 50 g of a sticky dark greenish material.

### Instruments and chromatographic conditions

Gas chromatography mass spectrography (GC-MS) analysis of *n*-hexane extract was carried out using GC-MS spectrometry instrument at Department of Medicinal and Aromatic Plants Research, National Research Centre. A TRACE GC ultra gas chromatograph (THERMO Scientific Corp., Waltham, MA, USA) is coupled with a thermo mass spectrometer detector (ISQ Single Quadrupole Mass Spectrometer). The GC-MS system was equipped with a TG-5MS column (30 m × 0.25 mm i.d., 0.25 μm film thickness). Helium is used as carrier gas at a flow rate of 1.0 mL/min and a split ratio of 1:10. Temperature programming is applied (50 °C for 3 min; rising at 5.0 °C/min to 300 °C and held for 20 min). The injector and detector were held at 280 °C. Diluted samples (1:10 hexane, v/v) of 0.2 μL were injected. Mass spectra were obtained by electron ionization (EI) at 70 eV, using a spectral range of *m*/*z* 40–450.

### Chemicals and kits

Paracetamol or acetaminophen (*N*-acetyl-*p*-aminophenol; APAP) was purchased from Sanofi-aventisegypts.a.e., El Sawah, El Amiriya, Cairo, Egypt. The kits used for biochemical measurements were all purchased from Bio-diagnostic Company, Dokki, Giza, Egypt. Other chemicals were of the highest purity commercially available.

### Experimental animals

This study was carried out at Zoology Department, Faculty of Science, Ain Shams University, using clinically healthy mature adult male Wistar albino rats. The animals were obtained from the Animal Breeding House of the National Research Centre (NRC), Dokki, Cairo, Egypt, and maintained in clea plastic cages in the laboratory animal house. Their weights ranged from 130 to 140 g. The rats were acclimatized for 1 week prior to the start of experiments on standard pellet diet (Agricultural-Industrial Integration Company, Giza, Egypt), tap water *ad libitum*, and daily 12 h dark/light cycle. All animals were humanely treated in accordance with World Health Organization (WHO, 1998) guideline for animal care and the study design was approved by the Ain Shams University Research Ethics Committee. The animals were accommodated in the laboratory conditions for one week before beginning the experiment.

### Acute toxicity study of the extract

The acute toxicity test was conducted to determine the median lethal dose (LD_50_) of test materials and used to choose the safe dose of the experiment for the *A. fraxinifolius* extract. The doses were selected based on acute toxic class method for Organization for Economic Cooperation and Development guidelines (OECD 423). The rats were divided into six groups. Group I received distilled water only as a vehicle while Groups II, III, IV, V and VI acted as treatment groups receiving single increasing dose of *A. fraxinifolius* extract at 1, 2, 3, 4 and 5 g/kg b.w of rats, respectively. All the doses and vehicle were administered by the oral route. Throughout the study period, all animals were observed for behavioural signs of toxicity, morbidity and mortality. All signs of these were noted after 1, 4 and 24 h of administration of the extract for 14 consecutive days. Finally, 1/20 (250 mg/kg b.w) and 1/10 (500 mg/kg b.w) of the maximum safe dose of the *A*. *fraxinifolius* extract tested for acute toxicity were selected as two doses for the experimental models.

### Experimental design

Experimental animals were randomly divided into six groups of five rats each: three healthy groups and three APAP groups. In healthy groups, Group 1 (healthy control group) animals received orally and daily 1.0 mL distilled water as vehicle by stomach tube. In Group 2 (control treated with *A. fraxinifolius* 250), each animal received *A. fraxinifolius* in a single daily oral dose of 250 mg/kg b.w alone for seven days. In Group 3 (control treated with *A. fraxinifolius* 500), each animal received *A. fraxinifolius* in a single daily oral dose of 500 mg/kg b.w alone for seven days. In APAP groups, in Group 4 (APAP only), each animal received a single daily oral dose of 400 mg/kg b.w APAP for seven days. Hepatotoxicity was induced in rats according to the procedure described by Kanchana and Mohamed (2011). In Group 5 (APAP + *A. fraxinifolius* 250), each animal received a single daily low dose of *A. fraxinifolius* orally starting 1 h before administration APAP and given for seven days. In Group 6 (APAP + *A. fraxinifolius* 500), each animal was given a single daily high dose of *A. fraxinifolius* orally starting 1 h before administration APAP and given for seven days.

After 24 h from the last treatment, the rats were humanely sacrificed by light diethyl ethyl ether anesthesia. The blood was collected into clean test-tubes without anticoagulant. Then, blood was centrifuged in a cooling centrifuge (IEC centra-4R; International Equipment Co., Needham Heights, MA) for 30 min at 3000 rpm and 4 °C to separate the clot from the serum in whole blood specimens. The serum was separated and divided into samples and preserved at –80 °C for further analysis. Immediately after killing the animals, livers of each animal were separately weighed and homogenized in 5 mL cold buffer (0.5 g of Na_2_HPO_4_ and 0.7 g of NaH_2_PO_4_ per 500 mL deionized water, pH 7.4) per gram tissue. Then, the homogenates were centrifuged for 15 min at 4000 rpm and 4 °C; and the obtained supernatants were divided into samples and preserved at –80 °C until used for evaluating the lipid peroxidation and total antioxidant parameters.

### Biochemical analysis

Body-weight gain or loss was calculated by using a Sartorius LP2200S balance (Gottingen, Germany) as the following equation: body-weight gain or loss = b.w at the end of the experiment –  b.w at the beginning of the experiment. All biochemical analyses were manually done using commercial kits. Determination of amino transaminases enzymes (AST and ALT), ALP activities, total protein, albumin, total and direct bilirubin, total cholesterol, triglycerides and HDL-cholesterol in serum was carried out as described by Reitman and Frankel (1975), Belfield and Goldberg (1971), Gornal et al. (1949), Dumas (1971), Walter and Gerade (1970), Richmond (1973), Fossati and Prencipe (1982) and Burstein et al. (1970), respectively. Malondialdehyde (MDA) and total antioxidant capacity in liver homogenate were estimated by the spectrophotometric method described by Ohkawa et al. (1979) and Koracevic et al. (2001), respectively. Serum total globulins and indirect bilirubin concentrations were calculated according to the following equations: total globulins = serum total protein – albumin; indirect bilirubin = total bilirubin – direct bilirubin. In addition, A/G ratio = albumin: globulin. LDL-cholesterol concentration was calculated according to the equation of Friedewald et al. (1972): LDL-cholesterol = total cholesterol – (TAG/5) – HDL-cholesterol. Atherogenic indexes were calculated as follows: atherogenic index (1) = total cholesterol : HDL-cholesterol ratio; atherogenic index (2) = LDL-cholesterol : HDL-cholesterol ratio.

### Statistical analysis

Data are presented as mean values with their standard errors. Statistical analysis was performed with analysis of variance (ANOVA), and the differences among groups were determined by Tukey’s multiple comparison test (Turner & Thayer 2001) using Graph Pad Prism version 4.03 for Windows (GraphPad software Inc., San Diego, CA). *p* values of <0.05, <0.01 and <0.001 were considered statistically significant, highly significant and very highly significant, respectively.

## Results

### Investigation of the components of *n*-hexane extract of *A. fraxinifolius* using GC-MS analysis

**Figure 1.**
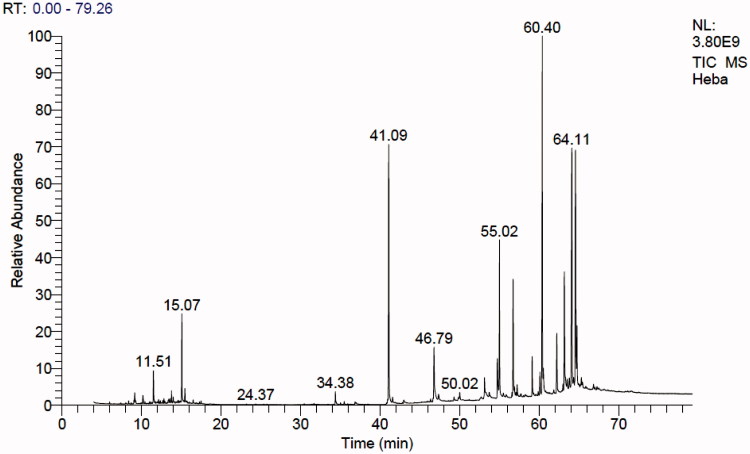
GC-MS chromatogram of *n-*hexane extract of *A. fraxinifolius* leaves.

The result of the GC-MS analysis of *n*-hexane extract of *A. fraxinifolius* leaves is shown in Figure 1 and Table 1. It revealed the presence of 37 compounds from which 29 compounds (95.9%) were identified. Hydrocarbons (acyclic and cyclic) represented 14.10%, steroids; 9.82%, triterpenes; 12.47% and other oxygenated compounds; 59.42%. α-Tocopherol was the major identified compound (18.23%) followed by labda 8 (20)-13-dien-15-oic acid (13.15%), lupeol (11.93%), phytol (10.95%), and squalene (7.19%). Identification of the compounds was carried out by matching their retention times and fragmentation patterns with those of reference compounds analysed under the same conditions (Selim 2013; Abdelwahab 2009; AOCS Lipid Library 2014).

**Table 1.** Identification of the components of *n-*hexane extract of *A. fraxinifolius* leaves.

No.	Compound^a^	Molecular formula	Molecular weight	Rt (min.)	Relative area (%)^c^	KI^b^	Method of identification
1	Unidentified	–	–	10.19	0.56	–	–
2	Undecane	C_11_H_24_	156	11.51	1.44	1099	KI, MS
3	Undecane, 5-methyl-	C_12_H_26_	170	13.42	0.36	1158	KI, MS
4	Undecane, 2methyl-	C_15_H_32_	212	13.77	0.61	1168	KI, MS
5	Undecane, 3-methyl-	C_12_H_26_	170	14.00	0.46	1172	KI, MS
6	Dodecane	C_14_H_30_	198	15.07	3.43	1200	KI, MS
7	Undecane, 2,6-dimethyl-	C_13_H_28_	184	15.46	0.65	1216	KI, MS
8	Neophytadiene	C_20_H_38_	278	34.38	0.70	1830	KI, MS
9	Palmitic acid methyl ester	C_17_H_34_O_2_	270	36.87	0.31	1920	KI, MS
**10**	**Phytol**	**C_20_H_40_O**	**296**	**41.09**	**10.95**	**1949**	KI, MS
11	Methyl stearate	C_19_H_38_O_2_	298	41.57	0.39	2099	KI, MS
12	Hexadecanamide	C_16_H_33_NO	255	42.98	0.37	2150	KI, MS
13	Oleamide	C_18_H_35_NO	281	46.79	2.70	2375	KI, MS
14	Unidentified	–	–	47.36	0.45	–	–
15	1,2-Benzenedicarboxylic acid, bis(2-ethyl hexyl) ester	C_24_H_38_O_4_	390	50.02	0.51	2510	KI, MS
16	Unidentified	–	–	53.14	1.05	–	–
17	Methyl tetracosanoate	C_21_H_42_O_4_	358	53.74	0.41	2732	KI, MS
18	16-Hydroxyhexadecanoic acid	C_16_H_32_O_3_	272	54.76	2.28	2737	KI, MS
19	**Squalene**	**C_30_H_50_**	**410**	**55.02**	**7.19**	**2806**	KI, MS
20	Unidentified	–	–	55.51	0.33	–	–
21	Unidentified	–	–	56.59	0.31	–	–
22	*n*-Tetracosanol-1	C_24_H_50_O	354	56.73	5.46	2905	KI, MS
23	Unidentified	–	–	56.91	0.35	–	–
24	Hexacosanoic acid, methyl ester-	C_27_H_54_O2	410	57.24	0.67	2941	KI, MS
25	γ-Tocopherol	C_27_H_46_O2	402	59.13	1.96	2987	KI, MS
26	Unidentified	–	–	59.89	0.32	–	–
27	**Vitamin E**	**C_29_H_50_O_2_,**	**430**	**60.40**	**18.23**	**3112**	KI, MS
28	α-Tocopherolquinone	C_29_H_50_O_3_	446	60.55	0.77	3113	KI, MS
29	Campesterol	C_28_H_48_O	400	61.85	0.37	3305	K, MS
30	Stigmasta-5,22-dien-3-ol (3á,22E)	C_29_H_48_O	412	62.23	3.01	3316	KI, MS
31	Unidentified	–	–	63.00	0.48	–	–
32	**γ-Sitosterol**	**C_29_H_50_O**	**414**	**63.18**	**5.86**	**3408**	KI, MS
33	Friedelin	C_30_H_50_O	426	63.53	0.54	2416	KI, MS
34	Unidentified	–	–	63.81	0.81	–	–
35	**Labda8(20),13-dien-15-oic acid (E)-(+)-**	**C_20_H_32_O_2_**	**304**	**64.11**	**13.15**	**2422**	KI, MS
36	33-Norgorgosta-5,24(28)-dien-3-ol,(3á)	C_29_H_46_O	410	64.35	0.58	–	MS
**37**	**Lupeol**	**C_30_H_50_O**	**427**	**64.57**	**11.93**	–	MS

a Compounds are listed in order of their elution

b Kovats retention index calculated on DB-5 column

c Average of three analyses

MS: identification based on mass spectral data; RI: identification based on comparison with published retention indices in Wiley Registry of Mass Spectral Data, 8th edition, mainlib Library, replib Library, NIST Mass Spectral Library (December 2005) and other published data. The major components are highlighted in bold.

### Acute toxicity of *A. fraxinifolius*

Acute toxicity study in which the animals treated with *A. fraxinifolius* extracts at a higher dose of 5000 mg/kg did not manifest any significant abnormal signs, behaviour changes or macroscopic findings at any time of observation, hence the doses of 250 and 500 mg/kg were taken for the study.

### Modulatory effects of *A. fraxinifolius* on body weight loss and liver relative weight

**Figure 2.**
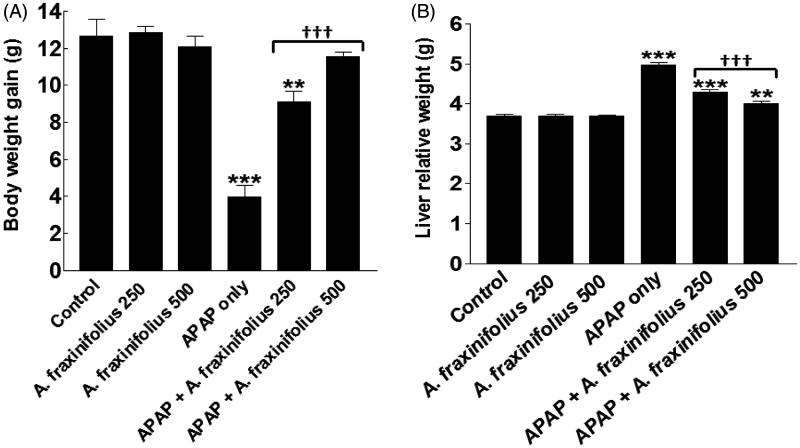
Body weight gain (A) and liver relative weight (B) of control and intoxicated rats. Values are means, with their standard errors represented by vertical bars. *A. fraxinifolius*: *Acrocarpus fraxinifolius*; APAP: *N*-acetyl-*p*-aminophenol **p < 0.01, ***p < 0.001: compared with the healthy control group; †††*p* < 0.001: compared with the APAP-intoxicated group that received vehicle; (one-way ANOVA with Tukey’s multiple comparison test).

Body weight gain and liver relative weight were significantly decreased and increased (*p*< 0.001) by –68.5% and 34.9%, respectively, in APAP-intoxicated group compared with the healthy control rats (Figure 2(A, B)). Both doses of *A. fraxinifolius* significantly alleviated (*p <* 0.001) the decrease and increase in the body weight gain and liver relative weight, respectively, of APAP- intoxicated rats during the treatment course. The body weight change was reverted to normal levels upon treatment with high dose of *A. fraxinifolius* (*p* > 0.05 compared with the healthy control group). In addition, the improvement of liver relative weight of APAP rats by high dose of *A. fraxinifolius* significantly exceeded (*p* < 0.01) that of low dose of *A. fraxinifolius.* The percentages of changes of the body weight gain and liver relative weight measured in the APAP-only-treated group and groups treated with APAP plus either the low or high dose of *A. fraxinifolius* were –68.5% (*M* = 3.98), –27.8% (*M* = 9.12), –8.4% (*M* = 11.58), 34.9% (*M* = 4.98), 16.3% (*M* = 4.29) and 8.7% (*M* = 4.01), respectively, compared with healthy control group.

### Modulatory effects of *A. fraxinifolius* on hepatocyte integrity markers

**Figure 3.**
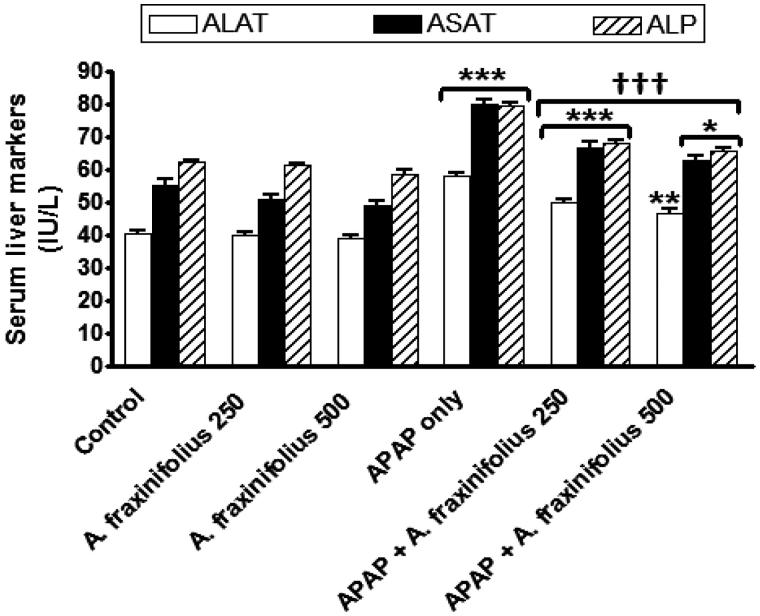
Serum liver enzymes markers of control and intoxicated rats. Values are means, with their standard errors represented by vertical bars. *A. fraxinifolius*: *Acrocarpus fraxinifolius*; ALAT: alanine aminotransferase; ALP: *alkaline phosphatase*; APAP: *N*-acetyl-*p*-aminophenol; ASAT: aspartate aminotransferase *p < 0.05, **p < 0.01,***p < 0.001: compared with the healthy control group; †††*p* < 0.001: compared with the APAP-intoxicated group that received vehicle; (one-way ANOVA with Tukey’s multiple comparison test).

As shown in Figure 3, APAP administration significantly increased (*p* < 0.001) serum ALAT, ASAT and ALP activities by 42.6% (*M* = 58.05), 44.5% (*M* = 80.24) and 27.8% (*M* = 79.51), respectively, compared with the healthy control group. Oral treatment of both doses of A*. fraxinifolius* significantly decreased (*p* < 0.001) serum ALT, AST and ALP activities compared with APAP-intoxicated group. The highest suppressive effect on the decrease on serum hepatocyte integrity markers of APAP rats was induced by the high dose of *A. fraxinifolius* (14.8%, 13.2% and 6.0%, respectively, compared with healthy control group). The percentages of changes of the serum ALAT, ASAT and ALP activities measured in the groups treated with APAP plus either the low or high dose of *A. fraxinifolius* were 22.6% (*M* = 49.88) vs 14.8% (*M* = 46.74), 19.9% (*M* = 66.6) vs 13.2% (*M* = 62.87) and 9.9% (*M* = 68.36) *vs* 6.0% (*M* = 65.96), respectively, compared with healthy control group.

### Modulatory effects o*f A. fraxinifolius* on serum lipid profiles

**Figure 4.**
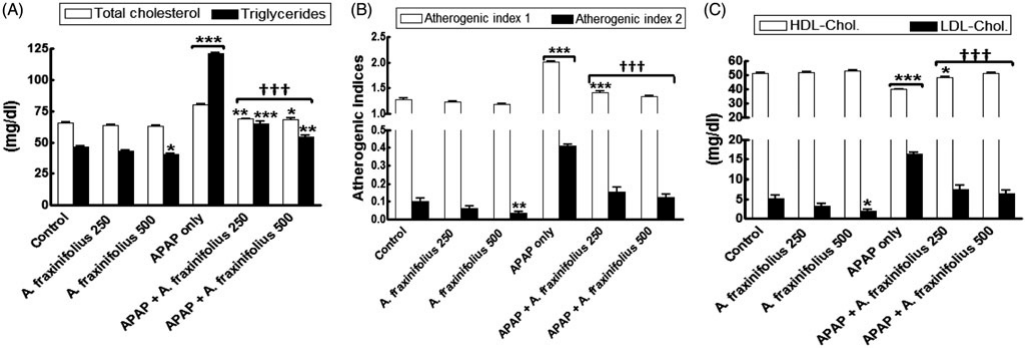
Serum lipid profiles (A: total cholesterol and triglycerides, B: HDL-cholesterol and LDL-cholesterol, C: atherogenic index 1 and 2) of control and intoxicated rats. Values are means, with their standard errors represented by vertical bars. *A. fraxinifolius*: *Acrocarpus fraxinifolius*; APAP: *N*-acetyl-*p*-aminophenol Atherogenic index (1), total cholesterol: HDL-cholesterol ratio; atherogenic index (2), LDL-cholesterol: HDL-cholesterol ratio *p < 0.05, **p < 0.01, ***p < 0.001: compared with the healthy control group; †††*p* < 0.001: compared with the APAP-intoxicated group that received vehicle; (one-way ANOVA with Tukey’s multiple comparison test).

Figure 4 revealed that healthy rats consumed high dose of *A. fraxinifolius* showed a significant decrease in the serum triglycerides, LDL-cholesterol and atherogenic index 1 (*p* < 0.05 to *p* < 0.01) by –12.5% (*M* = 40.73), –61.9% (*M* = 1.99) and –7.3% (*M* = 1.19), respectively, compared with the healthy control animals. The significant increase in serum total cholesterol, triglycerides, LDL-cholesterol and atherogenic indices (*p* < 0.001) by 22.6% (*M* = 80.46), 160.2% (*M* = 121.10), 213.2% (*M* = 16.33), 56.9% (*M* = 2.02) and 298.4% (*M* = 0.41), respectively, as shown in APAP-intoxicated group compared with the healthy control animals. On the other hand, serum HDL-cholesterol significantly decreased (*p* < 0.001) by –21.9% (*M* = 39.91) in APAP-intoxicated group compared with the healthy control rats. Both doses of *A. fraxinifolius* significantly alleviated (*p* < 0.05–0.001) the changes in the lipid profiles of APAP rats during the treatment course. These changes in LDL-cholesterol, HDL-cholesterol and atherogenic indices were reverted to normal levels upon treatment with both doses of *A. fraxinifolius* (*p* > 0.05 compared with the healthy control group), but the utmost modulation on the changes in serum lipid profiles were shown in APAP groups that received high doses of *A. fraxinifolius.* The percentages of changes of the serum total cholesterol, triglycerides, HDL-cholesterol, LDL-cholesterol and atherogenic indices measured in the groups treated with APAP plus either the low or high dose of *A. fraxinifolius* were 4.8% (*M* = 68.81) vs 4.4% (*M* = 68.54), 39.4% (*M* = 64.92) vs 16.3% (*M* = 54.14), –5.3% (*M* = 48.41) vs 51.29% (*M* = 0.31), 42.3% (*M* = 7.42) vs 23.1% (*M* = 6.42), 10.7% (*M* = 1.42) vs 4.0% (*M* = 1.34) and 50.4% (*M* = 0.15) vs 21.9% (*M* = 0.13), respectively, compared with healthy control group.

### Modulatory effects of *A. fraxinifolius* on serum protein profiles

**Figure 5.**
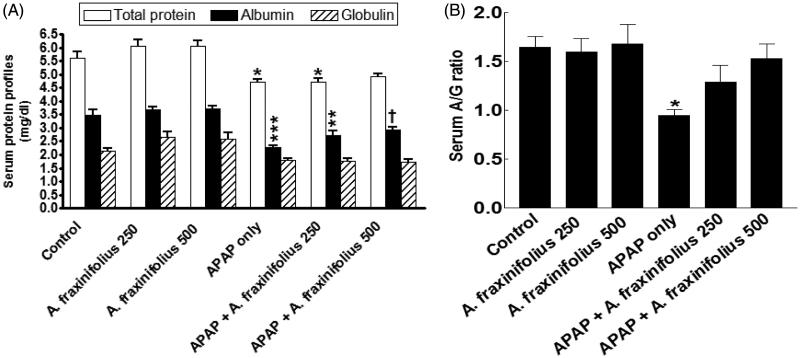
Serum total protein, albumin and globulin levels (A) and A/G ratio (B) of control and intoxicated rats. Values are means, with their standard errors represented by vertical bars. *A. fraxinifolius*: *Acrocarpus fraxinifolius*; APAP: *N*-acetyl-*p*-aminophenol *p < 0.05, **p < 0.01, ***p < 0.001: compared with the healthy control group; †*p* < 0.05: compared with the APAP-intoxicated group that received vehicle; (one-way ANOVA with Tukey’s multiple comparison test).

Serum the total protein, albumin levels and A/G ratio significantly increased (*p* < 0.05 to *p* < 0.01) by –16.1% (*M* = 4.72), –34.8% (*M* = 2.28) and –42.7% (*M* = 0.94), respectively, but serum globulin did not significantly alter (*p* > 0.05) by –16.6% (*M* = 1.78) in APAP-intoxicated group compared with the healthy control animals (Figure 5). Only 500 mg/kg of *A. fraxinifolius* completely modulated all the changes in serum protein profiles shown in APAP groups (*p* > 0.05 or *p* > 0.05) compared with the healthy control/APAP-intoxicated groups, respectively. The percentages of changes of serum total protein, albumin, globulin levels and A/G ratio, compared with the healthy control group, in APAP groups that received either 250 or 500 mg/kg of *A. fraxinifolius*, were –16.2% (*M* = 4.71) vs –12.7% (*M* = 4.91), –22.5% (*M* = 2.71) vs –16.1% (*M* = 2.93), –17.8% (*M* = 1.76) vs –19.4% (*M* = 1.72) and –21.9% (*M* = 1.29) vs –7.3% (*M* = 1.53), respectively, compared with the healthy control animals; indicating that the high dose of *A. fraxinifolius* exceeded that of low dose in APAP rat model.

### Modulatory effects of *A. fraxinifolius* on serum bilirubin level

**Figure 6.**
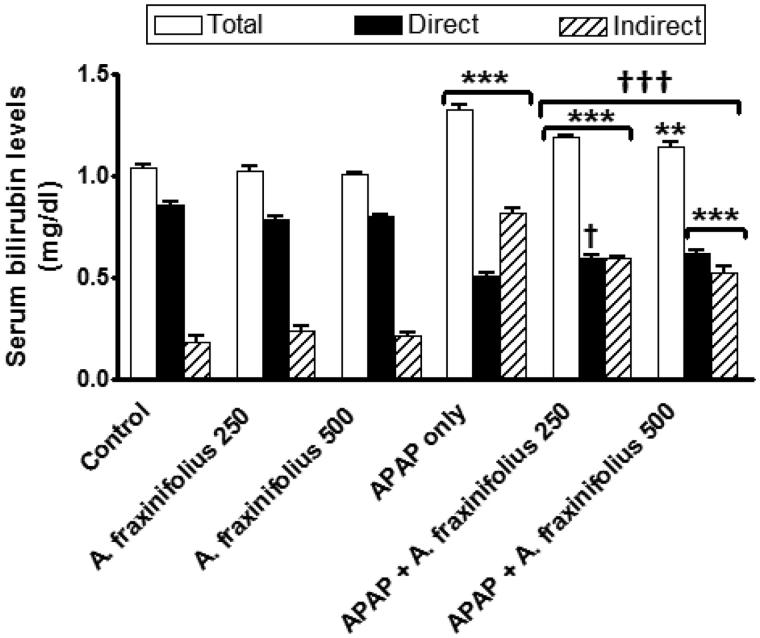
Serum total, direct and indirect bilirubin levels of control and intoxicated rats. Values are means, with their standard errors represented by vertical bars. *A. fraxinifolius*: *Acrocarpus fraxinifolius*; APAP: *N*-acetyl-*p*-aminophenol **p < 0.01, ***p < 0.001: compared with the healthy control group; †*p* < 0.05, †††*p* < 0.001: compared with the APAP-intoxicated group that received vehicle; (one-way ANOVA with Tukey’s multiple comparison test).

Figure 6 reveals that the significant increase in serum total, direct and indirect bilirubin levels (*p* < 0.001) by 27.7% (*M* = 1.33), –40.4% (*M* = 0.51) and 348.4% (*M* = 0.82), respectively, as shown in APAP control toxicity group compared with the healthy control animals. Oral treatment of both doses of *A. fraxinifolius* significantly decreased (from *p* < 0.05 to *p* < 0.001) serum total, direct and indirect bilirubin levels compared with APAP control toxicity group. The modulatory effects of high dose of *A. fraxinifolius* on serum total, direct and indirect bilirubin levels were more efficacious than that of low dose of *A. fraxinifolius*. The percentages of changes of serum total, direct and indirect bilirubin levels, compared with the healthy control group, in APAP groups that received either low or high dose of *A. fraxinifolius* were 14.4% (*M* = 1.19) vs 10.6% (*M* = 1.15), –30.6% (*M* = 0.59) vs –27.3% (*M* = 0.62) and 226.4% (*M* = 0.59) vs 189.0% (*M* = 0.53), respectively, compared with the healthy control animals.

### Modulatory effects of A. fraxinifolius on hepatic oxidative stress markers

**Figure 7.**
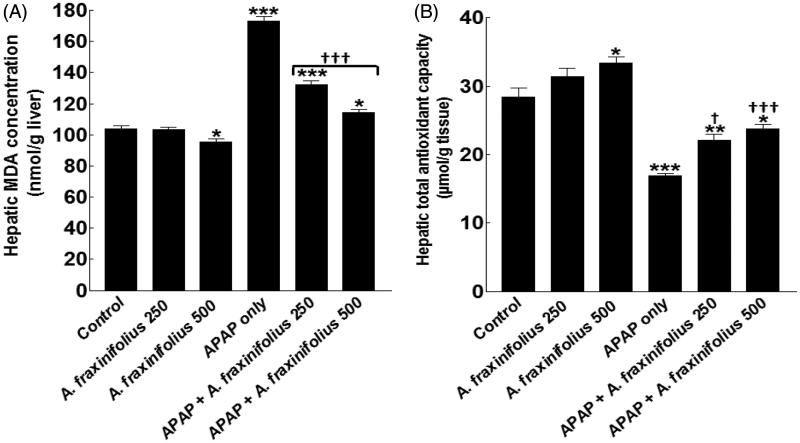
Hepatic MDA concentration (A) and total antioxidant capacity (B) of control and intoxicated rats. Values are means, with their standard errors represented by vertical bars. *A. fraxinifolius*: *Acrocarpus fraxinifolius*; APAP: *N*-acetyl-*p*-aminophenol; MDA: malondialdehyde *p < 0.05, **p < 0.01, ***p < 0.001: compared with the healthy control group; †*p* < 0.05, †††*p* < 0.001: compared with the APAP-intoxicated group that received vehicle; (one-way ANOVA with Tukey’s multiple comparison test).

Healthy rats that consumed high dose of *A. fraxinifolius* showed a significant decrease/increase (*p* < 0.05) in the hepatic MDA and total antioxidant levels by –8.5% (*M* = 95.21) and 17.4% (*M* = 33.33), respectively, compared with the healthy control animals (Figure 7). The hepatic MDA and total antioxidant levels significantly increased/decreased (*p* < 0.01) by 66.5% (*M* = 173.30) and –40.4% (*M* = 16.94), respectively, in APAP-intoxicated group compared with the healthy control animals. Both doses of *A. fraxinifolius* significantly alleviated (from *p* < 0.05 to *p* < 0.001) hepatic MDA and total antioxidant levels compared with APAP-intoxicated group. In addition, the improvement of hepatic MDA and total antioxidant levels of APAP rats by high dose of *A. fraxinifolius* significantly exceeded that of low dose of *A. fraxinifolius*. The percentages of changes of hepatic MDA and total antioxidant levels, compared with the healthy control group, in APAP groups that received either 250 or 500 mg/kg of *A. fraxinifolius* were 27.3% (*M* = 132.5) vs 9.70% (*M* = 114.20), and –22.1% (*M* = 22.13) vs –16.3% (*M* = 23.78), respectively, compared with the healthy control animals

All other parameters measured in this study were not significantly altered (*p* > 0.05) in healthy rats that received either low or high dose of *A. fraxinifolius* compared with the healthy control animals. Moreover, the mortality rates were zero in all groups treated with *A. fraxinifolius*. Therefore, no deleterious effects were detected for the dose of *A. fraxinifolius* used in this study.

## Discussion

Liver injury induced by APAP was widely used as a model for the screening and studying of hepatoprotective drugs as well as herbal therapies. Other studies demonstrated that hepatocytes are targeted by APAP (Slattery et al. 1987; Gazzani et al. 1989). This study demonstrated that APAP significantly elevated liver relative weight, the serum hepatic enzymes markers, lipid profiles and bilirubin. These results were mainly due to increase and decrease the hepatic MDA concentration and total antioxidant capacity, respectively. In addition, APAP decreased the body weight and serum protein profile compared with the control animals. Elevated lipid peroxidation causes degradation of cellular macromolecules that was concomitant with a reduction of antioxidant system leading to damage of several tissues (Basu et al. 2012). Also, Basu et al. (2012) reported that elevation serum cholesterol and triglyceride levels in APAP treated rats indicated impaired fat metabolism due to hepatic damage. The serum activity of ALAT and ASAT was used as a biochemical marker for early acute liver necrosis. Approximately 5% of the metabolites of APAP is normally converted into NAPQI (toxic agent responsible for the hepatotoxicity) through the action of CYP P450 system in the liver but during overdosing of APAP, detoxification of NAPQI is limited because of insufficient non-enzymic anti-oxidant system resulting from their depletion (Kaplowitz 2004; Geiger & Howard 2007; Josephy 2005). Serum protein and bilirubin levels are related to the function of the hepatocytes revealing the functional status of the liver. Hepatotoxicity may directly result either from protein damage or by the accumulation of reactive oxygen and nitrogen species (ROS and NOS) (James et al. 2003). All of the above parameters indicating the presence of oxidative injury were markedly reversed by *A. fraxinifolius* treatment, suggesting that *A. fraxinifolius* has a potent anti-oxidant effect on APAP hepatotoxicity. Our data indicate that GC-MS analysis of *n*-hexane extract of A. fraxinifolius leaves revealed the presence of five major compounds: α-tocopherol (18.23%), labda-8 (20)-13-dien-15-oic acid (13.15%), lupeol (11.93%), phytol (10.95%) and squalene (7.19%). This study found that both doses of *A. fraxinifolius* significantly alleviated the hepatotoxicity of APAP by alleviating the serum hepatocyte integrity markers (ALAT, ASAT and ALP activities), lipid contents, protein and bilirubin levels through increasing/decreasing the production of hepatic total antioxidant capacity/lipid peroxidation, respectively, and increasing the body weight gain. In addition, this study proves the hepatoprotective activity of 500 mg/kg of *A. fraxinifolius* over 250 mg/kg of *A. fraxinifolius*, which has not been established before.

α-Tocopherol is an isoform of vitamin E which has a powerful antioxidant activity in detoxifying free radicals, stabilization of the cell membrane and structure restoration (Sodhi et al. 2008; Banudevi et al. 2006). Along with our results, α-tocopherol was reported to reduce the elevated hepatic marker enzymes, lipid peroxidation and improve the oxidative damages of liver (Palipoch et al. 2014). Furthermore, α-tocopherol stimulated the upregulation of endogenous CYP3A4 and CYP3A5 which metabolize APAP into reactive metabolite NAPQI (Landes et al. 2003). Also Kothekar et al. (2004) showed that α-tocopherol was effective in reversing the hepatotoxicity induced by isoniazid, rifampicin and pyrazinamide combination.

In addition, α-tocopherol is more potent than β-carotene and ascorbic acid in combating ethanol induced hepatic oxidative stress (Datta et al. 2012). Labdane-type diterpenes isolated from Brazilian propolis extract also exerted a significant hepatoprotective activity (Banskota et al. 2008). Lupeol, a pentacyclic triterpenes, was reported to improve the antioxidant status of the liver in cadmium intoxicated rats (Sunitha et al. 2001). The antioxidant and hepatoprotective activities exerted by *Ficus pseudopalma* Blanco (Moraceae) extract against paracetamol-induced oxidative damage was attributed to lupeol (Arimado & Santiago 2015). Lupeol, isolated from the petroleum ether extract of *Diospyros cordifolia* Roxb (Ebenaceae) stem bark, exhibited significant hepatoprotective activity against carbon tetrachloride induced toxic hepatitis (Mankani et al. 2006). Lupeol, exhibited protective effect against 7,12-dimethylbenz(*a*)anthracene (DMBA) induced hepatotoxicity (Prasad et al. 2008). Regarding squalene Sivakrishnan and Muthu (2014) and Zuhan et al. **(**2015) reported about the promising hepatoprotective effects of squalene isolated from *Albizia procera* (Roxb.) Benth (Mimosaceae) against APAP and CCl_4_-induced toxicity. Phytol, which is an acyclic diterpene alcohol, acts as a precursor for vitamin E and K1 and it has antioxidant and anticancer activities (Oyugi et al. 2011; Serafini et al. 2011). So, these reports confirmed the hepatoprotective activity of *n*-hexane extract of *A. fraxinifolius* which was attributed to presence of mainly α-tocopherol and terpenoidal compounds. In general, *A. fraxinifolius* is considered to be safe herbal medicine with insignificant adverse/side effects on healthy male rats. The low and high doses of *A. fraxinifolius* used in this study are equivalent to 2.4 and 4.8 g/day/human and did not induce any deleterious effects in healthy rats.

## Conclusion

In conclusion*, n*-hexane extract of *A. fraxinifolius* having anti-hepatotoxic properties may reduce the harmful effects generated by APAP-induced hepatotoxicity by preventing lipid peroxidation, and through its modulation on total antioxidant capacity. The alleviating effects of *n*-hexane extract of *A. fraxinifolius* on some parameters measured in this study were partial, but significant, and dose dependent. Subsequently, it may be more useful if the dose or time increased in future studies.
